# Application of SNV Detection Methods for Market Control of Food Products from New Genomic Techniques

**DOI:** 10.3390/ijms27020626

**Published:** 2026-01-08

**Authors:** Klaudia Urszula Bernacka, Krzysztof Michalski, Marek Wojciechowski, Sławomir Sowa

**Affiliations:** Plant Breeding and Acclimatization Institute—National Research Institute, Radzików, 05-870 Błonie, Poland; k.bernacka@ihar.edu.pl (K.U.B.); krzysztof.michalski@ihar.edu.pl (K.M.); m.wojciechowski@ihar.edu.pl (M.W.)

**Keywords:** single-nucleotide variants (SNV), genetically modified organism (GMO), new genomic techniques (NGT), food/feed market control

## Abstract

The detection of single-nucleotide variants (SNVs) is an important challenge in modern genomics, with broad applications in medicine, diagnostics, and agricultural biotechnology. Current detection approaches include PCR-based techniques with high-affinity probes, ligase-based strategies, and sequencing approaches, each with varying degrees of sensitivity, specificity, and practicality. Despite advances in SNV analysis in the medical field, their implementation in the official control and monitoring of genetically modified organisms (GMOs) remains limited. This challenge has gained priority with the advent of new genomic techniques (NGTs), such as CRISPR-Cas nucleases, which allow precise genome editing, including subtle changes at the nucleotide level without introducing foreign DNA. Therefore, traditional methods of GMO detection targeting transgene sequences may not be sufficient to monitor such GMOs. In the European Union, GMO legislation requires distinguishing between conventionally bred and genetically modified plants. The planned introduction of new regulatory categories of NGT plants (NGT1 and NGT2) with different surveillance requirements emphasizes the need for robust, sensitive, and cost-effective SNV detection methods suitable for distinguishing between GMOs, particularly in the context of food and feed safety, traceability, and compliance.

## 1. Introduction

The detection of SNVs is a well-established analytical approach widely used in medicine, diagnostics and agrobiotechnology [1,2,3]. SNVs, together with insertions and deletions, account for the majority of genetic variation between individuals. Besides SNV, the term SNP (single-nucleotide polymorphism) is also commonly used. A “variant” refers to any single-nucleotide change, regardless of its origin or frequency of occurrence. An SNP, on the other hand, refers to the germline (meaning it is heritable) and must be present in at least 1% of the population. Nevertheless, from a detection perspective, these changes are equivalent [4,5,6,7]. Therefore, in this review, all single-nucleotide substitutions are collectively referred to as SNVs.

A broad range of SNV detection methods have been developed, including PCR-based assays with high-affinity probes, ligation-based strategies, and sequencing approaches [8,9,10,11,12]. These techniques differ in sensitivity, specificity, cost, and complexity, and while they are routinely applied in medical diagnostics, their use outside the field remains limited. In plant breeding, SNV detection is mainly employed for marker-assisted selection, and, until recently, there was little need to adapt such methods for official GMO control [13,14,15,16,17,18,19].

Current EU GMO legislation is based on the detection of exogenous DNA elements introduced by transgenesis and requires analytical methods to meet defined minimum performance requirements (MPR), including robustness, specificity, and limit of detection [15,20]. The current strategy for the detection and identification of specific events involves performing screening, followed by event-specific analysis. The preliminary test seeks commonly used foreign genetic elements (e.g., the 35S promoter delivered from the cauliflower mosaic virus). There are 27 DNA screening elements that enable the detection of both authorized GMOs (currently 104 events for which specific methods are available) and unauthorized GMOs (for which the GM event-specific methods are unavailable) [21,22,23,24,25]. However, plants obtained through genome editing often only contain small nucleotide-level changes without foreign DNA. This challenges conventional GMO detection strategies and highlights the need for sensitive SNV-based methods that are suitable for market control of food and feed.

The proposed regulatory framework identifies two categories of NGT plants. NGT category 1 (NGT1) plants may contain up to 20 genetic modifications with a maximum size of 20 nucleotides each (excluding deletions which are not size restricted). Plants in this category are considered equivalent to conventionally bred; thus, the restrictions applied to traditional GMOs will not apply to them, but labeling their reproductive material as an NGT1 product is still planned. On the other hand, NGT category 2 (NGT2) are plants that do not meet NGT1 criteria, and the same restrictions will be applied to them as to conventional GMOs (with an easier authorization procedure in case of traits, with a positive impact on a sustainable agrifood system) [20,23,26,27,28]. As a result, we can predominantly expect gene edited plants with only minor changes to be placed on the market.

Oversight of genome editing in food and feed is governed by the European Food Safety Authority (EFSA) [22,29,30,31,32], within the framework of EU legislation, including Regulation (EC) No 1829/2003 [22] establishing authorization and labeling requirements, and Regulation (EU) No 619/2011 [25] defining analytical procedures and thresholds for unauthorized GM material.

This review aims to critically evaluate current SNV detection methods with respect to their applicability in official GMO and NGT control. The spotlight is placed on analytical performance parameters required by EU legislation (limit of detection (LOD), limit of quantification (LOQ), specificity, robustness, and quantification capability), and on identifying methodological gaps that limit the implementation of SNV-based approaches for market surveillance of NGT1 and NGT2 plants [20,23,26].

## 2. State of the Art in Point Mutation Detection

### 2.1. Methods Based on Target Sequence Amplification

For many years, SNV assays have been used in crop plants for variety identification and for detecting genetic markers associated with desirable traits. These include restriction fragment length polymorphism (RFLP), random amplified polymorphic DNA (RAPD), real-time PCR, and amplified fragment length polymorphism (AFLP), which were primarily applied for qualitative analyses or zygosity determination in the 0–0.5–1 system [13,14,16,17,18,19]. Historically, there has been no demand for quantitative SNV analyses in either GMOs or plants in general.

In contrast, numerous PCR-based SNV detection methods have been developed in medical diagnostics. Many of them simply rely on target amplification, but polymerase tolerance to primer–template mismatches can result in false positives [33,34,35]. To improve specificity and reliability, alternative approaches based on sequence-specific probes, ligation assays, or CRISPR/Cas systems have been developed [33,36,37,38,39].

#### 2.1.1. Applications of Specific Probes

##### LNA Probes

Single-nucleotide variant detection frequently relies on oligonucleotides composed of nucleic acid analogues, used either as PCR probes/primers or as polymerase blockers [40]. Among these, locked nucleic acid (LNA) probes are the most widely applied due to their enhanced mismatch discrimination. The presence of a methylene bridge (2′-O,4′-C) increases duplex stability and binding energy, resulting in a markedly higher melting temperature difference between perfectly matched and mismatched hybrids, which may reach 20–30 °C for a single base mismatch [40].

As a result, LNA probes provide a relatively simple, cost-effective, and highly specific approach for SNV detection using PCR-based techniques, while also enabling quantitative analysis. Optimal performance depends on probe design, including oligonucleotide length (18–24 bases) and the central placement of LNA residues, with approximately half of the bases substituted by LNA [41]. This design enables the detection of DNA variants at ratios as low as 0.003% [42].

**Figure 1:**
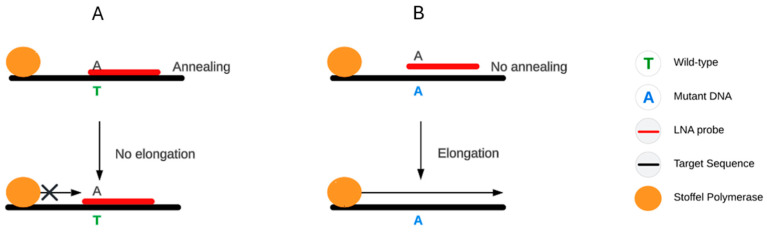
Schematic of LNA as a blocking probe. Stoffel polymerase is devoid of 5′-3′ exonuclease activity; therefore, it can be blocked by a hybridized probe, stopping amplification (**A**); mismatch preventing annealing results in sequence extension (**B**).

LNA oligonucleotides can also be applied to selectively suppress amplification of wild-type alleles (Figure 1) [40]. Novel LNA-based approaches continue to be developed, such as toehold-mediated strand-displacement reactions, which improve sensitivity in terms of DNA concentration but offer limited improvement in LOD [43]. LNA probe tendency to form secondary structures necessitates the use of dedicated in silico design tools, including the IDT SciTools OligoAnalyzer, for reliable primer and probe optimization [44,45].

##### PNA Probes

Peptide Nucleic Acid (PNA), another nucleic acid analogue, is used in a similar manner to LNA. Its backbone is composed of a chain of peptide-like N-(2-aminoethyl) glycine. PNA oligonucleotides exhibit higher melting temperatures as a result of a lack of electrical charge.

The analysis by Fouz and Apella [46] showed higher binding specificity of PNA probes for SNV detection. Assays were based on differences in melting temperature which, depending on the site and type of mismatch, reached 8–20 °C. The PNA oligonucleotides were also used in droplet digital PCR (ddPCR) as allele selective polymerization blockers, increasing sensitivity from 0.25% to 0.1% [47].

Unlike other oligonucleotide analogues, this modification prevents degradation by nucleases but is more expensive and lowers specificity. Consequently, the use of PNAs with short sequences is not advised for detecting mutations below 1% of SNV content, as they may yield false positive or false negative results [47].

##### OWL and MB Probes

**Figure 2:**
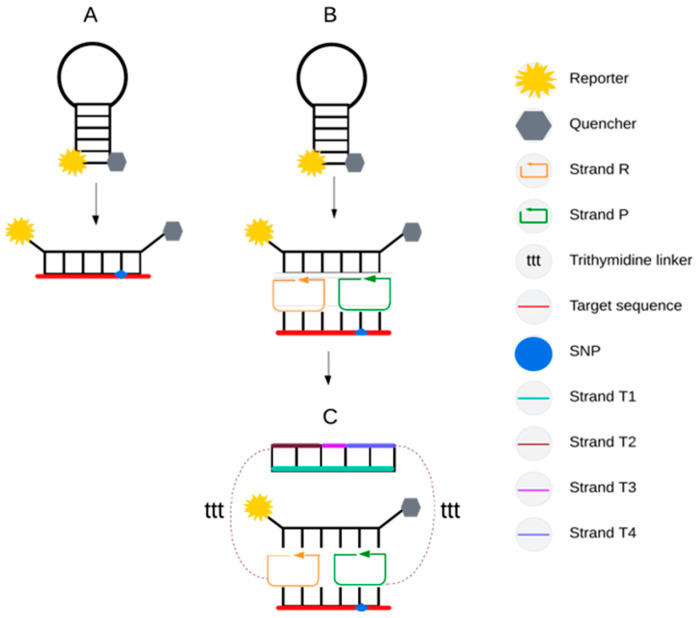
Schematic representation of assays based on MB; (**A**) probe is opened upon binding, as a result of template complementarity. In the case of OWL1 (**B**), the strand R and strand P oligonucleotides open the MB probe structure, with one of the strands targeting SNV of interest. Both strands fold into circular forms at the 3′ and 5′ ends, forming a structural lock, which increases sensitivity to mismatches. The sensor uses DNA as the building block of a nanostructure that is unstable in the presence of a single-nucleotide mismatch. The P and R strands are target specific while the MB probe does not hybridize directly to DNA [48,50]. OWL2 (**C**) was modified by providing a secondary structure, consisting of scaffolding (T1), one fragment complementary to the R strand (T3), and two fragments containing long template-binding arms (T2 and T4). This complex system quenches fluorescence in case of a mismatch and opens the MB probe (in case of a perfect match) on the principle of “stretching” the probe on two sides by T2 and T4 [48,50].

Other SNV detection approaches include OWL (OWL1 and OWL2) and molecular beacon (MB) probes (Figure 2A–C). These methods are based on primer binding in the presence of a universal molecular beacon (UMB), leading to the formation of an “OWL” nucleic acid structure. MB probes are GC-rich, which increases melting temperature and fluorescence quenching efficiency by bringing the reporter and quencher into close proximity. Upon hybridization with a complementary target, the hairpin structure opens, generating a fluorescence signal (Figure 2A). Unlike PCR, detection is performed during an annealing step at low temperatures (5–32 °C) for up to 60 min [33,48,49,50,51].

In OWL1, primers induce opening of the MB structure, with one oligonucleotide containing a target specific base. The resulting circularized oligonucleotide configuration creates a structural lock that enhances mismatch discrimination (Figure 2B). Detection selectivity depends not only on probe–target duplex stability but also on the stability of the entire nucleic acid nanostructure, which fails to assemble in the presence of a single-nucleotide mismatch. A key advantage of OWL probes is their modular design, enabling simplified optimization and multiplexing, as target specificity is determined by primers rather than by direct probe–DNA hybridization [48,50].

OWL2 was developed to improve sensitivity and specificity by introducing an additional scaffold and extended template-binding arms, which stretch the UMB probe only in the presence of a perfectly matched target (Figure 2C) [48,50]. This approach demonstrates enhanced performance compared with OWL1, including successful SNV detection, although both OWL variants remain unsuitable for quantitative analysis [50].

#### 2.1.2. Application of Ligases

##### OLA

**Figure 3:**
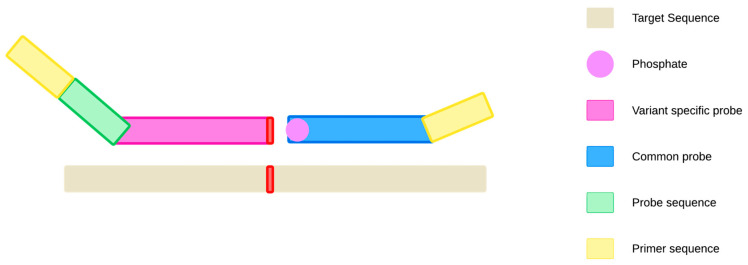
Schematic representation of OLA. The assay requires the use of two ligation oligonucleotides: common (phosphorylated and complementary to both wild-type and variant DNA sequences) and variable (containing the variant specific base of interest at the end of 3′). DNA ligase covalently joins the two probes only if both are perfectly hybridized to their target templates, including the variant base. Both ligation probes contain adapters, which later allow for amplification of the ligation product with complementary PCR primers. The variable probe adapter also contains a hybridization site for the fluorescently tagged qPCR probe [8,52].

The oligonucleotide ligation assay (OLA) was specifically developed for the determination of single-nucleotide polymorphisms and is a technique that is based on nucleic acid hybridization. The assay requires the simultaneous use of both common and variable ligation probes (Figure 3).

DNA ligase covalently joins the two oligonucleotides only if both are perfectly hybridized to their target templates, including the altered base [8,52]. Multiplexing is possible by attaching different fluorophores to probes, or by changing sequence lengths on allele-specific probes (thus changing the Tm) [8,36,52]. The lengths of flanking regions range from 15 bp to 30 bp. However, as a rule of thumb, the greater the length of the flank, the lower the selectivity. In addition, the variable probes targeting should have different melting temperatures, for higher specificity. The specificity of the assay is impacted by the SNV adjacent sequence, and consequently also by the choice of ligase itself.

Upon successful optimization, OLA can be combined with qPCR and allows for high sensitivity to be achieved qualitatively as well as quantitatively. Additionally, OLAgen software was developed to facilitate the design of ligation probes. OLA achieved a sensitivity of 1%; however, lower mutated copy content was not attempted [8,53].

##### LCR

Ligase chain reaction (LCR) employs four primers, two per DNA strand, which hybridize adjacently to form a ligation junction that is sealed only in the absence of a mismatch, enabling SNV discrimination. High specificity is achieved through mismatch-sensitive ligation, provided that blunt-end ligation activity is minimized to prevent target-independent ligation events [9]. LCR allows both qualitative and quantitative analysis and can be adapted for multiplexing; however, its specificity decreases when the proportion of variant alleles is low relative to the wild-type sequence [9,54]. Reported limits of detection are typically around 0.1% [54].

**Figure 4:**
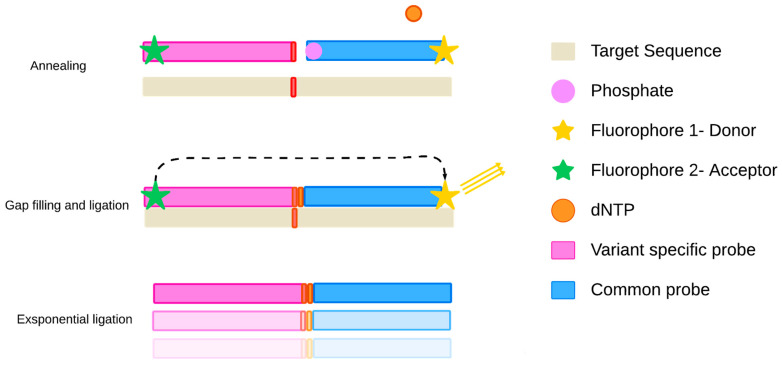
Schematic representation of assay based on QGLCR, which is a combination of Gap-LCR and qPCR. The main steps of this method are probe annealing, gap filling by polymerase, and subsequent ligation of probes. By default, the ligation will occur only in the presence of a target variant, as polymerase cannot fill the gap without the corresponding nucleotide substrate. The whole process can be monitored in real-time through fluorescence transfer between the fluorophores of labeled oligonucleotides upon their ligation. The excited energy state of the first fluorophore is transferred to the second one, which is followed by light emission at its characteristic spectrum window [55].

An advanced variant, quantitative gap-LCR (QGLCR), combines Gap-LCR with qPCR and real-time fluorescence readout (Figure 4) [9,54]. In this approach, a single-nucleotide gap between adjacent probes is filled by DNA polymerase, followed by ligation. Only the nucleotide corresponding to the target variant base is added to the reaction mixture. Using probe lengths of 15–16 nt, QGLCR can achieve limits of detection as low as 0.001%, although higher values (~0.085%) have also been reported [9,55,56]. A major limitation of this approach remains the difficulty of implementing robust multiplex designs [55,56].

##### LAMP

Loop-mediated isothermal amplification (LAMP) is well suited for analysis of samples with low DNA input and relies on *Bst* DNA polymerase, which enables rapid strand-displacement amplification without the need for thermocycling [57,58]. Due to its high efficiency and minimal equipment requirements, LAMP is particularly attractive for routine qualitative analyses in laboratories with limited technical resources [59,60,61]. However, conventional LAMP exhibits limited specificity for detecting single-nucleotide variants [57].

**Figure 5:**
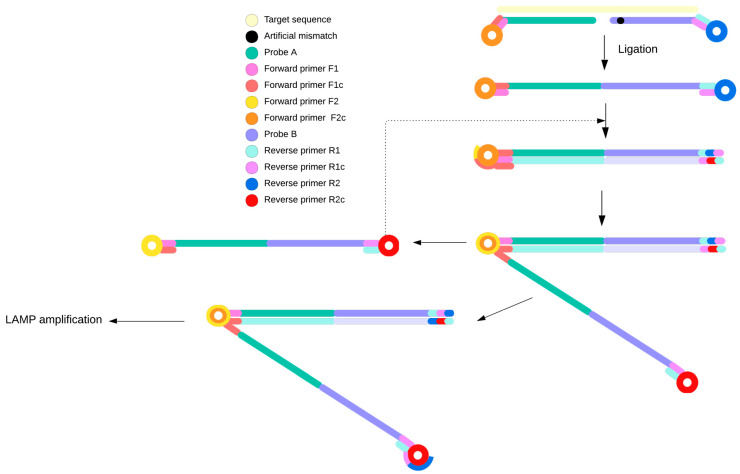
Schematic representation of assay based on AML-LAMP. Two ligation oligonucleotides hybridize adjacently to a single-stranded DNA template, and DNA ligase joins them into a continuous strand only when their ends are perfectly matched to the target sequence. An artificially introduced mismatch, upstream to the target SNV, increases allele discrimination at the ligation step. This mismatch destabilizes probe–template hybridization when the target base is not complementary, thereby preventing ligation. Upon successful ligation, the resulting molecule forms a stem-loop structure, which is then recognized by LAMP primers and extended by DNA polymerase, initiating isothermal DNA amplification [58].

Specificity can be improved by introducing artificial mismatches, as implemented in artificial mismatched ligation–LAMP (AML-LAMP). This approach uses two probes: a universal phosphorylated probe and a mutation-specific probe containing an additional 3′-end mismatch to suppress nonspecific ligation (Figure 5). Ligation occurs only in the presence of the target variant, followed by standard LAMP, which can be monitored in real time using intercalating fluorescent dyes. Reported limits of detection for AML-LAMP are approximately 0.01%, while the method is generally considered suitable for qualitative rather than quantitative analysis [58].

LAMP assays may incorporate colorimetric detection using pH-sensitive dyes or metallic nanoparticles [10,62,63]. Although such approaches enable visual interpretation without instrumentation, they are prone to false-positive signals and are not compatible with multiplex analysis, limiting their applicability in routine testing [10].

##### RCA

The isothermal rolling circle amplification (RCA) is frequently used for specific amplification in diagnostic tests [63,64,65,66]. In addition to primers and dNTPs, RCA requires a strand displacement polymerase (e.g., *Phi29*) and padlock probes.

**Figure 6:**
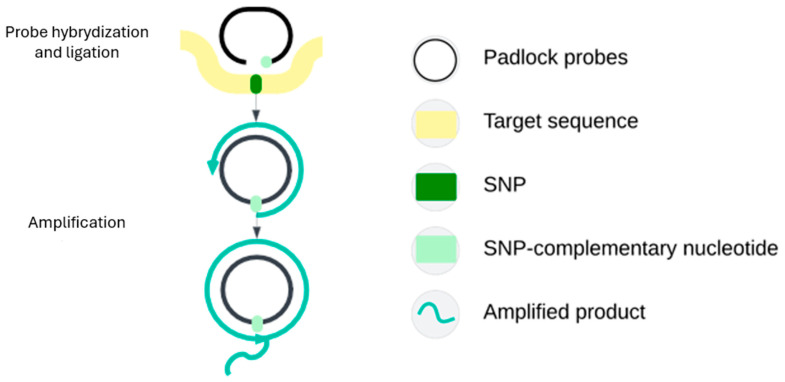
Schematic representation of assay based on L-RCA. Initially, a padlock probe hybridizes to the target DNA sequence in such a way that its 5′ and 3′ ends are directly adjacent to each other on the template. If the sequence match is correct, DNA ligase joins the ends of the probe to form a closed, circular DNA molecule. Next, a primer is attached to the circular probe, and a strand displacement polymerase begins DNA synthesis, repeatedly circling the closed probe. This process results in a very long single-stranded DNA product containing numerous repeats of a template sequence [69].

RCA can be used in two ways. The first, stand-alone RCA, uses the target DNA as a primer to amplify a previously closed padlock probe [64,67]. The second, more common, is ligation RCA (L-RCA), which uses the DNA template to close the padlock probe by ligation, forming a circular DNA structure that can be amplified. Probe closure occurs only if both ends hybridize perfectly (Figure 6).ariants of RCA include branched RCA (BRCA) and hyper-branched RCA (HRCA). BRCA uses an additional primer that binds to the RCA product and initiates synthesis of a new DNA strand, forming branched structures that increase detection sensitivity. HRCA follows the same principle but requires more primers [67,68,69,70].

Readouts include fluorescence increase in real-time or gel electrophoresis [66]. RCA products can be further analyzed quantitatively, e.g., by real-time PCR [64,71].

#### 2.1.3. Application of CRISPR-Cas

Cas nuclease specificity is determined by guide RNA molecules. Their cleavage sites are located 2–3 nucleotides up-stream of the protospacer adjacent motif (PAM), a short (2-6nt) Cas specific sequence [72]. The effectors (Cas9, Cas12, Cas13) depend on the target sequence, as they recognize different PAMs and substrates: Cas9 and Cas12 bind and cleave dsDNA, while Cas13 targets RNA [73].

The integration of CRISPR-Cas nucleases with other methods has led to techniques such as Cas-EXPAR (exponential amplification reaction), RPA (recombinase polymerase amplification), SHERLOCK (Specific High-Sensitivity Enzymatic Reporter UnLOCKing), HOLMES (one-HOur Low-cost Multipurpose highly Efficient System) and others [73,74,75,76,77]. Cas-EXPAR combines CRISPR/Cas9 precision with the sensitivity of isothermal EXPAR, where the Cas9/sgRNA complex cuts target DNA to initiate amplification [78]. HOLMES uses Cas12a to detect DNA after amplification, employing non-specific nuclease activity for signal generation [76]. SHERLOCK uses Cas13a ribonuclease activity to degrade reporter RNA molecules following isothermal target amplification and transcription, achieving an LOD of 0.1% [73,74].

CRISPR/Cas12a- or Cas13a-based RCA can detect targets within amplified RCA products. RCA product is recognized by the Cas12a–crRNA complex through template-guide complementarity. Resulting activation of the complex triggers the non-specific trans-nuclease activity of Cas12a, cutting single-stranded DNA reporters present in the solution and generating a measurable fluorescent signal. This approach enables multiplexing and very sensitive detection, with reported LODs as low as 0.036% [79,80], which, while slightly higher than some LNA probe-based methods, remains highly effective for diagnostic applications.

### 2.2. Sequencing-Based Methods

SNVs can be detected directly by DNA sequencing, with all its generations commonly used for this purpose [81]. While highly precise, sequencing is labor-intensive and relatively expensive. SNVs can be identified via whole-genome sequencing (WGS) or targeted sequencing, with the latter being more commonly used due to lower costs.

Sequencing read length typically ranges from 150 to 300 bp, depending on the method. GC-rich or GC-poor sequences can introduce errors during library preparation and sequencing, especially in PCR amplification due to polymerase low fidelity. Errors can be mitigated by optimizing polymerases, thermocycling conditions, and buffers [82]. High-fidelity polymerases (e.g., Q5, Phusion, Platinum Taq) and minimizing amplification cycles help reduce errors and preserve library complexity [11].

#### 2.2.1. Sanger

Sanger sequencing, a first-generation technology, relies on the incorporation of chain-terminating ddNTPs by DNA polymerase during amplification. Sanger sequencing is commonly used for routine analysis of a small number of targets due to its low cost and simplicity compared to next generation sequencing (NGS). It is unsuitable for quantitative analysis, requiring homogeneous samples and having a theoretical detection limit of 15–20% [83,84]. Poor performance can result from low template quality or quantity, heterogeneous samples, or sequence complexity (e.g., GC-rich or repetitive). Chromatogram analysis is important to verify sequencing quality and sample homogeneity [85].

#### 2.2.2. Illumina

Sequencing technology developed by Illumina reads short DNA fragments. Illumina‘s sequencing by synthesis (SBS) uses fluorescently labeled nucleotides and reversible terminators, which both contribute to its high accuracy and massively parallel sequencing capabilities [86]. Library preparation involves DNA fragmentation (physical or enzymatic), end repair, and adapter ligation for PCR amplification and flow cell attachment. Illumina is a gold standard in NGS, with multiple library reagent suppliers [87]. Sequencing is robust to errors due to sample heterogeneity (99.2% specificity), and no differences in specificity towards specific substitutions (W↔S; transversions) were observed. The number of reads and depth of coverage have a key impact on the accuracy of Illumina sequencing. Low coverage increases the risk of false negatives. Conversely, too-high coverage does not guarantee a reduction in false positives—they may even increase in certain regions [88]. Fortunately, these errors can often be corrected at the data analysis stage. In summary, SNV detection based on Illumina technology achieves a LOD of 0.1% [12].

#### 2.2.3. AVITI

**Figure 7:**
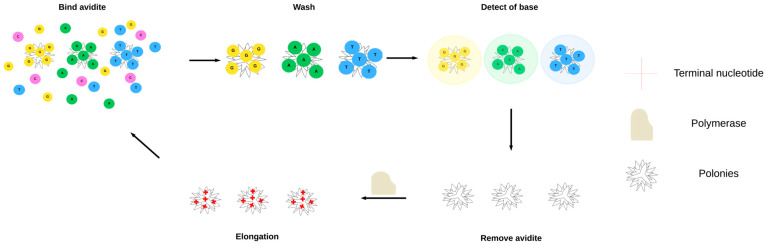
Schematic representation of assay based on AVITI.

AVITI is a next-generation SBS platform that generates sequence data via binding of avidites: multivalent, labeled oligonucleotide ligands. This approach differs from the likes of Illumina and Ion Torrent, where the sequencing signal is generated simultaneously to nucleotide incorporation during synthesis. First, the sequencing polonies are generated on a solid base, using rolling-circle amplification (Figure 7). Then the sequencing reaction proceeds as follows: (i) polymerase molecules bind to the 3′ ends of the multiple primer-template complexes in the polony and select the complementary avidites, allowing a fluorescence readout; (ii) avidites are removed from the polonies; (iii) template strand is extended by one nucleotide, and the cycle can be repeated [89]. Such simultaneous interaction with multiple sites increases the binding affinity 100-fold over an individual interaction. This method promises a reduction in errors and an increase in variant detection sensitivity, potentially making it an improvement over Illumina sequencing for SNV diagnostics [90,91,92,93]. Additional research would be required to verify the applicability of this platform for SNV detection [91].

#### 2.2.4. Ion Torrent

Ion Torrent is a next-generation semiconductor chip-based sequencing technology. To generate the sequencing template, the library is clonally amplified by emulsion PCR onto beads. These beads are then placed in millions of wells connected to a semiconductor chip. Amplified DNA fragments serve as templates and are sequenced by sequentially introducing unlabeled and unmodified nucleotides in a base-by-base order. Nucleotide incorporation and the creation of a phosphodiester bond leads to a surge of protons, causing shifts in pH and electric potential, which are then detected by semiconductor chips. Since detection is direct, each nucleotide inclusion is recorded within seconds [12]. If a nucleotide is not incorporated, no voltage change is registered, which enables a simple sequence readout [94]. This technology tends to generate a lot of false positives in indel detection. Ion Torrent and Illumina have been repeatedly compared due to them generating similar error types. It has been shown that the frequency of these errors is much higher with Ion Torrent. However, upon appropriate optimization (e.g., enhancement of coverage depth), it is possible to achieve satisfactory results, with a sensitivity of 0.15% and 100% accuracy [12,95].

#### 2.2.5. Long-Read Sequencing

Long-read sequencing (third-generation sequencing, e.g., PacBio, Oxford Nanopore Technologies), although highly useful for analyzing structural genomic variations, is not the optimal method for detecting SNVs in food and feed. Firstly, it suffers from a higher per-read error rate compared to short-read technologies [38]. Secondly, the fragmentation of template DNA in processed material renders the biggest advantage of long-read sequencing–the length of reads, ineffective. Thirdly, calling variants from mixed samples requires a high coverage of the investigated site, once again making short read sequencing more efficient for this application. Nonetheless, we predict that third-generation sequencing will have its niche for calling SNVs in homogenous samples, especially when there is little known about the possible nature of tested sites [96,97].

## 3. Perspectives for Adaption to GMO Control

The results reported by various independent research groups were obtained for detecting human DNA or pathogen-specific SNVs. Despite satisfactory results, these techniques may not be directly applicable in GMO enforcement laboratories for detecting SNVs in plants and food/feed products. Plant and food samples are often heterogeneous, and processed foods may contain DNA mixtures from various species, risking unwanted amplification or interference. Additionally, enzyme inhibitors such as polyphenols, tannins, starch, and lipids can reduce PCR or isothermal method efficiency, especially in seeds, further reducing SNV detection sensitivity and repeatability [98,99,100,101]. Plant DNA also has a high content of repetitive sequences and cultivar variability, potentially causing misattribution of nucleotide changes [102]. Limited information exists on these methods’ suitability for GMO analysis.

Many established methods lack sufficient characterization to meet EURL criteria, making adaptability uncertain (Table 1). Standard validation requirements include practicability, DNA concentration and integrity, robustness, Relative Standard Deviation of Reproducibility (RSDr), and false positive/negative rates. However, LOD and LOQ definitions differ between the fields: in medicine, LOD refers to DNA concentration in the reaction mixture [50], while in GMOs it refers to modified DNA copy number [20], reflecting differences in material availability. Thus, the adequacy of these techniques needs to be tested empirically. However, based on their known drawbacks and LODs, it is possible to delineate methods in which optimization may prove to be a Sisyphean task. Such methods, due to their high LOD, include PNA (Table 1) [47]. In this context, the use of CRISPR/Cas has not been excluded, as it can be integrated with others [79]. Quantitative analysis is not possible with published LAMP methods and with OWL1 and MB probes, which is a significant drawback in the context of official control [50,59]. Nevertheless, LAMP appears to be a suitable choice for screening, despite even the somewhat more complicated ‘initial’ procedure. It is overly optimistic to assume that all techniques could be multiplexed, which is important for high-throughput analysis [58]. In the case of RCA and OLA, the amount of information is limited, but they seem to be promising tools to increase the specificity of any novel methods [52,69].

Nonetheless, some methods have been tested for GMO and NGT analyses. Among probe-based approaches, nucleic acid analogs have been used to improve existing PCR-based assays. For example, a qPCR method with LNA primers targeting *FAD2-1A* and FAD2-*1B* variants in soybean showed promising results, with in-house validation achieving an LOD of 3.7 copies and meeting other performance criteria. Interlaboratory validation confirmed acceptable performance, though the LOD increased to 11.2 copies, likely due to plasmid-based sample variability. Overall, the results were comparable to routine methods for conventional GMOs [103].

A CRISPR/Cas12a-based method for NGT rice detection was optimized to identify SNVs at the 12-copy level, but required a specific PAM [104]. This limitation can be addressed by using modified Cas proteins, such as SpRY (PAM-less Cas9 variant), which expand target range and enable precise single-nucleotide cleavage [105,106]. Combining SpRY with recombinase polymerase amplification (RPA) allowed for the detection of single-nucleotide substitutions at the 1% level in under one hour, offering a rapid, equipment-light screening tool, though it did not meet MPR [107].

Some studies evaluated the applicability of current SNV detection methods under official GMO control. A 2020 publication by Chhalliyil et al. [108] suggested quantification of NGT products using qPCR and Sanger sequencing, but replication attempts by Weidner et al. [109] revealed non-specific amplification and failure to meet MPR criteria, with quantitative analysis hindered by the absence of established performance standards.

Targeted high-throughput sequencing enables detection of genetically edited rice lines via unique SNV markers. The workflow involves whole-genome sequencing, comparative analysis to identify unique variants, and validation with targeted PCR and NGS. This method can detect gene content below 25 template copies per sample, offering high sensitivity, accuracy, and repeatability while meeting standards analogous to MPR and EURL, thus supporting effective monitoring even at very low edited-line content [20,110].

A reproducibility study in CRISPR/Cas9-edited oilseed rape compared targeted sequencing (Illumina) and WGS (PacBio Sequel II). Long-read technology showed higher interlaboratory variability than short reads, but targeted sequencing demonstrated sufficient sensitivity to detect GMOs at the 0.1% level [111].

A key challenge in adapting SNV detection methods for routine GMO and NGT testing is the lack of standardization and harmonization across laboratories. Even highly sensitive methods may yield non-comparable results due to differences in equipment, DNA extraction reagents, or data interpretation, affecting reproducibility [20,23]. Implementing SNV detection requires standards compatible with routine procedures, including uniform definition of method parameters, standardized bioinformatic analysis, and reporting [23,112,113]. Interlaboratory studies and proficiency tests help identify weaknesses, correct protocols, and set minimum requirements [20,112]. Harmonization is especially important for high-throughput or multiplexed analyses, since not all techniques integrate easily into single analytical workflows [114].

NGT regulations vary by modification type. NGT1 plants lack mandatory SNV detection validation, while NGT2 plants follow strict criteria like conventional GMOs (sensitivity, specificity, LOD/LOQ, repeatability) [20,23,26]. Regulatory gaps complicate differentiation between natural variation and genome-edited changes, highlighting the need to strengthen analytical capabilities [20,23,26].

## Figures and Tables

**Table 1:** Compared the properties of SNV detection techniques.

Basic Concept	Technique	Max. LOD	LOD in GMO	Application for Quantitative Analysis	Application for Multiplex Analysis	The Biggest Drawbacks
Based on probes	LNA [40,42]	0.003%	11.2 cp (plasmid)	+	+	Tendency to form secondary structures
	PNA [47]	0.1%	N/A	N/A	N/A	Low specificity
	OWL1/2 [48,50]	N/A	N/A	N/A	+	Binding to dsDNA Difficulty
	MB [48,50]	N/A	N/A	N/A	+	Low efficiency
Based on ligation	OLA [8,53]	<1%	N/A	+	+	Low specificity
	LCR [54]	0.1%	N/A	+	+	High LOD
	QGLCR [55]	0.001%	N/A	+	+	Validation
	LAMP [58]	0.01%	N/A	−/+	+	Validation
	RCA [69]	N/A	N/A	−/+	N/A	Difficulty
CRISPR/Cas-RCA [79]		0.036%	1%	+	+	Validation

## Data Availability

No new data were created or analyzed in this study.

## Glossary

Limit of detection (LOD)–The limit of detection is the lowest amount or concentration of target in a sample, which can be reliably detected, but not necessarily quantified.

Limit of quantification (LOQ)–The limit of quantification is the lowest amount or concentration of target in a sample that can be reliably quantified with an acceptable level of trueness and precision.

Detection–The ‘finding’ of a specific target sequence, i.e., detection sensu stricto, without necessarily being specific for the genome-edited event.

Identification–The possibility to unequivocally allocate the detected sequence to a specific GM event; this is equally applicable to conventional GMOs and to plants obtained by targeted mutagenesis or cisgenesis.

[20,21,22,23]

## References

[1] Kim S., Matsushita Y., Katagiri T., Maseda H. (2025). Efficiency of Genome Editing Using Modified Single-Stranded Oligodeoxyribonucleotides in Human Cells. Sci. Rep..

[2] Sahoo J., Mishra R., Joshi R.K. (2025). PCR-Based Single Nucleotide Polymorphism (SNP) Genotyping for Crop Improvement-Current Status and Future Prospects. Discov. Plants.

[3] Nesta A.V., Tafur D., Beck C.R. (2021). Hotspots of Human Mutation. Trends Genet..

[4] Yuan H., Liu W., Hu J., Zhang C. (2024). Recent Advances in Single-Nucleotide Variant Assay: From in Vitro Detection to in Vivo Imaging. Trends Anal. Chem..

[5] National Human Genome Research Institute Human Genomic Variation. https://www.genome.gov/about-genomics/educational-resources/fact-sheets/human-genomic-variation.

[6] Sayitoğlu M. (2016). Clinical Interpretation of Genomic Variations. Turk. J. Haematol..

[7] Willsey A.J. (2013). Single-Nucleotide Polymorphism. Encyclopedia of Autism Spectrum Disorders.

[8] Nelson D.J., Shilts M.H., Pakala S.B., Das S.R., Schmitz J.E., Haselton F.R. (2023). Ligation-Based Assay for Variant Typing without Sequencing: Application to SARS-CoV-2 Variants of Concern. Influenza Other Respir. Viruses.

[9] Gibriel A.A., Adel O. (2017). Advances in Ligase Chain Reaction and Ligation-Based Amplifications for Genotyping Assays: Detection and Applications. Mutat. Res. Rev. Mutat. Res..

[10] Lee J.-E., Mun H., Kim S.-R., Kim M.-G., Chang J.-Y., Shim W.-B. (2020). A Colorimetric Loop-Mediated Isothermal Amplification (LAMP) Assay Based on HRP-Mimicking Molecular Beacon for the Rapid Detection of *Vibrio parahaemolyticus*. Biosens. Bioelectron..

[11] Cheng C., Fei Z., Xiao P. (2023). Methods to Improve the Accuracy of Next-Generation Sequencing. Front. Bioeng. Biotechnol..

[12] Singh R.R. (2020). Next-Generation Sequencing in High-Sensitive Detection of Mutations in Tumors: Challenges, Advances, and Applications. J. Mol. Diagn..

[13] Celik I., Camci H., Kose A., Kosar F.C., Doganlar S., Frary A. (2016). Molecular Genetic Diversity and Association Mapping of Morphine Content and Agronomic Traits in Turkish Opium Poppy (*Papaver somniferum*) Germplasm. Mol. Breed..

[14] Chang M., Kim J.-Y., Lee H., Lee E., Lee W.-H., Moon S., Choe S., Choung C.M. (2022). Development of Diagnostic SNP Markers and a Novel SNP Genotyping Assay for Distinguishing Opium Poppies. Forensic Sci. Int..

[15] (2001). Directive 2001/18/EC of the European Parliament and of the Council of 12 March 2001 on the Deliberate Release into the Environment of Genetically Modified Organisms and Repealing Council Directive 90/220/EEC—Commission Declaration. http://data.europa.eu/eli/dir/2001/18/oj/eng.

[16] Ganal M.W., Altmann T., Röder M.S. (2009). SNP Identification in Crop Plants. Curr. Opin. Plant Biol..

[17] Gouda A.C., Warburton M.L., Djedatin G.L., Kpeki S.B., Wambugu P.W., Gnikoua K., Ndjiondjop M.N. (2021). Development and Validation of Diagnostic SNP Markers for Quality Control Genotyping in a Collection of Four Rice (Oryza) Species. Sci. Rep..

[18] Lin J.-J., Kuo J., Ma J., Saunders J.A., Beard H.S., MacDonald M.H., Kenworthy W., Ude G.N., Matthews B.F. (1996). Identification of Molecular Markers in Soybean Comparing RFLP, RAPD and AFLP DNA Mapping Techniques. Plant Mol. Biol. Rep..

[19] Sun J.-J., Xia X.-M., Wei X.-X., Wang X.-Q. (2023). Tracing the Geographic Origin of Endangered Plant Species Using Transcriptome-Derived SNPs: An Example of *Cathaya argyrophylla*. Mol. Ecol. Resour..

[20] European Network of GMO Laboratories (ENGL) (2015). JRC Technical Report—Definition of Minimum Performance Requirements for Analytical Methods of GMO Testing. https://gmo-crl.jrc.ec.europa.eu/doc/MPR%20Report%20Application%2020_10_2015.pdf.

[21] GMOMETHODS EURL GMFF. https://gmo-crl.jrc.ec.europa.eu/gmomethods/.

[22] (2003). Regulation (EC) No 1829/2003 of the European Parliament and of the Council of 22 September 2003 on Genetically Modified Food and Feed (Text with EEA Relevance). http://data.europa.eu/eli/reg/2003/1829/oj/eng.

[23] Gatto F., Savini C., Baillie C.-K., Broothaerts W., Burns M., Dagand E., Debode F., Dobnik D., Grantiņa-Ieviņa L., Grohmann L. (2023). Definition of Minimum Performance Requirements for Analytical Methods of GMO Testing—Part 2.

[24] Berben G., Boniotto B., Loose M., Garavelloni S., Grohmann L., Herau V., Holst-Jensen A., Hubert P., Narendja F., Onori R. (2017). JRC Technical Reports—Detection, Interpretation and Reporting on the Presence of Authorised and Unauthorised Genetically Modified Materials (Working Group Report).

[25] (2011). Commission Regulation (EU) No 619/2011 of 24 June 2011 Laying Down the Methods of Sampling and Analysis for the Official Control of Feed as Regards Presence of Genetically Modified Material for Which an Authorisation Procedure Is Pending or the Authorisation of Which Has Expired Text with EEA Relevance. http://data.europa.eu/eli/reg/2011/619/oj.

[26] (2023). Proposal for a Regulation of the European Parliament and of the Council on Plants Obtained by Certain New Genomic Techniques and Their Food and Feed, and Amending Regulation (EU) 2017/625. https://eur-lex.europa.eu/legal-content/EN/TXT/?uri=celex:52023PC0411.

[27] Cardi T., Murovec J., Bakhsh A., Boniecka J., Bruegmann T., Bull S.E., Eeckhaut T., Fladung M., Galovic V., Linkiewicz A. (2023). CRISPR/Cas-Mediated Plant Genome Editing: Outstanding Challenges a Decade after Implementation. Trends Plant Sci..

[28] Zhan X., Lu Y., Zhu J.-K., Botella J.R. (2021). Genome Editing for Plant Research and Crop Improvement. J. Integr. Plant Biol..

[29] (2003). Commission Regulation (EC) No 1304/2003 of 11 July 2003 on the Procedure Applied by the European Food Safety Authority to Requests for Scientific Opinions Referred to It (Text with EEA Relevance). http://data.europa.eu/eli/reg/2003/1304/oj/eng.

[30] (2004). Commission Regulation (EC) No 2230/2004 of 23 December 2004 Laying Down Detailed Rules for the Implementation of European Parliament and Council Regulation (EC) No 178/2002 with Regard to the Network of Organisations Operating in the Fields Within the European Food Safety Authority’s Mission. Text with EEA Relevance. http://data.europa.eu/eli/reg/2004/2230/oj/eng.

[31] (2002). Regulation (EC) No 178/2002 of the European Parliament and of the Council of 28 January 2002 Laying down the General Principles and Requirements of Food Law, Establishing the European Food Safety Authority and Laying down Procedures in Matters of Food Safety. http://data.europa.eu/eli/reg/2002/178/oj/eng.

[32] (2003). Regulation (EC) No 1642/2003 of the European Parliament and of the Council of 22 July 2003 Amending Regulation (EC) No 178/2002 Laying down the General Principles and Requirements of Food Law, Establishing the European Food Safety Authority and Laying down Procedures in Matters of Food Safety. http://data.europa.eu/eli/reg/2003/1642/oj/eng.

[33] Abi A., Safavi A. (2019). Targeted Detection of Single-Nucleotide Variations: Progress and Promise. ACS Sens..

[34] Stadhouders R., Pas S.D., Anber J., Voermans J., Mes T.H.M., Schutten M. (2010). The Effect of Primer-Template Mismatches on the Detection and Quantification of Nucleic Acids Using the 5′ Nuclease Assay. J. Mol. Diagn..

[35] Huang K., Zhang J., Li J., Qiu H., Wei L., Yang Y., Wang C. (2024). Exploring the Impact of Primer-Template Mismatches on PCR Performance of DNA Polymerases Varying in Proofreading Activity. Genes.

[36] Chauhan A., Toley B. (2024). Investigating the Compatibility of Polymerase Chain Reaction and Oligonucleotide Ligation Assay for One Step Detection of Point Mutations. ChemRxiv.

[37] Matsuda K. (2017). PCR-Based Detection Methods for Single-Nucleotide Polymorphism or Mutation: Real-Time PCR and Its Substantial Contribution Toward Technological Refinement. Adv. Clin. Chem..

[38] Wu K., Kong F., Zhang J., Tang Y., Chen Y., Chao L., Nie L., Huang Z. (2023). Recent Progress in Single-Nucleotide Polymorphism Biosensors. Biosensors.

[39] Yang N., Zhang H., Han X., Liu Z., Lu Y. (2024). Advancements and Applications of Loop-Mediated Isothermal Amplification Technology: A Comprehensive Overview. Front. Microbiol..

[40] Dominguez P.L., Kolodney M.S. (2005). Wild-Type Blocking Polymerase Chain Reaction for Detection of Single Nucleotide Minority Mutations from Clinical Specimens. Oncogene.

[41] Prout J., Tian M., Palladino A., Wright J., Thompson J.F. (2023). LNA Blockers for Improved Amplification Selectivity. Sci. Rep..

[42] Hashimoto Y., Masunaga N., Kagara N., Abe K., Yoshinami T., Tsukabe M., Sota Y., Miyake T., Tanei T., Shimoda M. (2023). Detection of Ultra-Rare ESR1 Mutations in Primary Breast Cancer Using LNA-Clamp ddPCR. Cancers.

[43] Wu F., Chen M., Lan J., Xia Y., Liu M., He W., Li C., Chen X., Chen J. (2017). A Universal Locked Nucleic Acid-Integrated X-Shaped DNA Probe Design for Amplified Fluorescence Detection of Single-Nucleotide Variant. Sens. Actuators B Chem..

[44] Oligo Analyzer. https://eu.idtdna.com/calc/analyzer.

[45] You Y., Moreira B.G., Behlke M.A., Owczarzy R. (2006). Design of LNA Probes That Improve Mismatch Discrimination. Nucleic Acids Res..

[46] Fouz M.F., Appella D.H. (2020). PNA Clamping in Nucleic Acid Amplification Protocols to Detect Single Nucleotide Mutations Related to Cancer. Molecules.

[47] Uchiyama Y., Nakashima M., Watanabe S., Miyajima M., Taguri M., Miyatake S., Miyake N., Saitsu H., Mishima H., Kinoshita A. (2016). Ultra–Sensitive Droplet Digital PCR for Detecting a Low–Prevalence Somatic GNAQ Mutation in Sturge–Weber Syndrome. Sci. Rep..

[48] Karadeema R.J., Stancescu M., Steidl T.P., Bertot S.C., Kolpashchikov D.M. (2018). The Owl Sensor: A ‘Fragile’ DNA Nanostructure for the Analysis of Single Nucleotide Variations. Nanoscale.

[49] Kolpashchikov D.M. (2012). An Elegant Biosensor Molecular Beacon Probe: Challenges and Recent Solutions. Scientifica.

[50] Mueller B.L., Liberman M.J., Kolpashchikov D.M. (2023). OWL2: A Molecular Beacon-Based Nanostructure for Highly Selective Detection of Single-Nucleotide Variations in Folded Nucleic Acids. Nanoscale.

[51] Tyagi S., Kramer F.R. (1996). Molecular Beacons: Probes That Fluoresce upon Hybridization. Nat. Biotechnol..

[52] Bui M.-H., Stone G.G., Nilius A.M., Almer L., Flamm R.K. (2003). PCR-Oligonucleotide Ligation Assay for Detection of Point Mutations Associated with Quinolone Resistance in Streptococcus Pneumoniae. Antimicrob. Agents Chemother..

[53] Nelson D.J., Chugh K., Pua H.H., Haselton F.R. (2025). A Software Tool for Reagent Design to Expand Access to Single-Nucleotide Variant Detection by the Oligonucleotide Ligation Assay. J. Mol. Diagn..

[54] Wang Z., Li L., Kuang Y., Yao J., Xu F., Chen Y. (2023). Simultaneous Quantification of Multiple Single Nucleotide Variants in PIK3CA ctDNA Using Mass-Tagged LCR Probe Sets. Talanta.

[55] Harden S.V., Thomas D.C., Benoit N., Minhas K., Westra W.H., Califano J.A., Koch W., Sidransky D. (2004). Real-Time Gap Ligase Chain Reaction: A Rapid Semiquantitative Assay for Detecting P53 Mutation at Low Levels in Surgical Margins and Lymph Nodes from Resected Lung and Head and Neck Tumors. Clin. Cancer Res..

[56] Psifidi A., Dovas C., Banos G. (2011). Novel Quantitative Real-Time LCR for the Sensitive Detection of SNP Frequencies in Pooled DNA: Method Development, Evaluation and Application. PLoS ONE.

[57] Kalofonou M., Malpartida-Cardenas K., Alexandrou G., Rodriguez-Manzano J., Yu L.-S., Miscourides N., Allsopp R., Gleason K.L.T., Goddard K., Fernandez-Garcia D. (2020). A Novel Hotspot Specific Isothermal Amplification Method for Detection of the Common PIK3CA p.H1047R Breast Cancer Mutation. Sci. Rep..

[58] Sun Y., Han B., Sun F. (2021). Ultra-Specific Genotyping of Single Nucleotide Variants by Ligase-Based Loop-Mediated Isothermal Amplification Coupled with a Modified Ligation Probe. RSC Adv..

[59] Grammatikos S., Svoliantopoulos I., Gizeli E. (2023). Naked-Eye Detection of LAMP-Produced Nucleic Acids in Saliva Using Chitosan-Capped AuNPs in a Single-Tube Assay. Anal. Chem..

[60] Costa-Junior L.M., Chaudhry U.N., Skuce P.J., Stack S., Sargison N.D. (2022). A Loop-Mediated Isothermal Amplification (LAMP) Assay to Identify Isotype 1 β-Tubulin Locus SNPs in Synthetic Double-Stranded Haemonchus Contortus DNA. J. Parasit. Dis..

[61] Khammanee T., Sawangjaroen N., Buncherd H., Tun A.W., Thanapongpichat S. (2021). A LAMP-SNP Assay Detecting C580Y Mutation in Pfkelch13 Gene from Clinically Dried Blood Spot Samples. Korean J. Parasitol..

[62] Brown T.A., Schaefer K.S., Tsang A., Yi H.A., Grimm J.B., Lemire A.L., Jradi F.M., Kim C., McGowan K., Ritola K. (2021). Direct Detection of SARS-CoV-2 RNA Using High-Contrast pH-Sensitive Dyes. J. Biomol. Tech..

[63] Garrido-Maestu A., Prado M. (2022). Naked-Eye Detection Strategies Coupled with Isothermal Nucleic Acid Amplification Techniques for the Detection of Human Pathogens. Compr. Rev. Food Sci. Food Saf..

[64] Garafutdinov R.R., Sakhabutdinova A.R., Gilvanov A.R., Chemeris A.V. (2021). Rolling Circle Amplification as a Universal Method for the Analysis of a Wide Range of Biological Targets. Russ. J. Bioorg Chem..

[65] Ouedraogo S.Y., Zeye M.M.J., Zhou X., Kiendrebeogo T.I., Zoure A.A., Chen H., Chen F., Ma C. (2024). Colorimetric Detection of Single-Nucleotide Mutations Based on Rolling Circle Amplification and G-Quadruplex-Based DNAzyme. Anal. Methods.

[66] Xiao J., Yuan C., Ding M., Hu W., Hu Z., Tian B. (2023). Photoacoustic Detection of SARS-CoV-2 Spike N501Y Single-Nucleotide Polymorphism Based on Branched Rolling Circle Amplification. Talanta.

[67] Ali M.M., Li F., Zhang Z., Zhang K., Kang D.-K., Ankrum J.A., Le X.C., Zhao W. (2014). Rolling Circle Amplification: A Versatile Tool for Chemical Biology, Materials Science and Medicine. Chem. Soc. Rev..

[68] Faruqi A.F., Hosono S., Driscoll M.D., Dean F.B., Alsmadi O., Bandaru R., Kumar G., Grimwade B., Zong Q., Sun Z. (2001). High-Throughput Genotyping of Single Nucleotide Polymorphisms with Rolling Circle Amplification. BMC Genom..

[69] Goo N.-I., Kim D.-E. (2016). Rolling Circle Amplification as Isothermal Gene Amplification in Molecular Diagnostics. BioChip J..

[70] Xu L., Duan J., Chen J., Ding S., Cheng W. (2021). Recent Advances in Rolling Circle Amplification-Based Biosensing Strategies-A Review. Anal. Chim. Acta.

[71] Gao T., Chai W., Shi L., Shi H., Sheng A., Yang J., Li G. (2019). A New Colorimetric Assay Method for the Detection of Anti-Hepatitis C Virus Antibody with High Sensitivity. Analyst.

[72] Zaidi S.S.-A., Mukhtar M.S., Mansoor S. (2018). Genome Editing: Targeting Susceptibility Genes for Plant Disease Resistance. Trends Biotechnol..

[73] Yan F., Wang W., Zhang J. (2019). CRISPR-Cas12 and Cas13: The Lesser Known Siblings of CRISPR-Cas9. Cell Biol. Toxicol..

[74] Gootenberg J.S., Abudayyeh O.O., Lee J.W., Essletzbichler P., Dy A.J., Joung J., Verdine V., Donghia N., Daringer N.M., Freije C.A. (2017). Nucleic Acid Detection with CRISPR-Cas13a/C2c2. Science.

[75] Huang M., Zhou X., Wang H., Xing D. (2018). Clustered Regularly Interspaced Short Palindromic Repeats/Cas9 Triggered Isothermal Amplification for Site-Specific Nucleic Acid Detection. Anal. Chem..

[76] Li S.-Y., Cheng Q.-X., Wang J.-M., Li X.-Y., Zhang Z.-L., Gao S., Cao R.-B., Zhao G.-P., Wang J. (2018). CRISPR-Cas12a-Assisted Nucleic Acid Detection. Cell Discov..

[77] Wang J., Cheng Q., Li S., Li X., Li L. (2021). Application of a Cas Protein, Method of Detection of a Target Nucleic Acid Molecule and Kit. https://patents.google.com/patent/EP3653722A4/en.

[78] Ou X., Li K., Liu M., Song J., Zuo Z., Guo Y. (2024). EXPAR for Biosensing: Recent Developments and Applications. Analyst.

[79] Cao G., Chen X., Deng Y., Nie F., Liu Y., Wang G., Huo D., Hou C. (2021). Single-Nucleotide Variant of PIK3CA H1047R Gene Assay by CRISPR/Cas12a Combined with Rolling Circle Amplification. Anal. Chim. Acta.

[80] Wu Y., Sun Y., Wang S., Wang X., Li K., Wang Y., Lu Z., Liu Q., Wang W., Dao Y. (2025). Development of a CRISPR/Cas12a One-Tube POCT Method with RCA for Multiplex Detection of SNPs. Microchem. J..

[81] Spencer D.H., Zhang B., Pfeifer J., Kulkarni S., Pfeifer J. (2015). Chapter 8—Single Nucleotide Variant Detection Using Next Generation Sequencing. Clinical Genomics.

[82] Head S.R., Komori H.K., LaMere S.A., Whisenant T., Van Nieuwerburgh F., Salomon D.R., Ordoukhanian P. (2014). Library Construction for Next-Generation Sequencing: Overviews and Challenges. BioTechniques.

[83] Al-Shuhaib M.B.S., Hashim H.O. (2023). Mastering DNA Chromatogram Analysis in Sanger Sequencing for Reliable Clinical Analysis. J. Genet. Eng. Biotechnol..

[84] Tsiatis A.C., Norris-Kirby A., Rich R.G., Hafez M.J., Gocke C.D., Eshleman J.R., Murphy K.M. (2010). Comparison of Sanger Sequencing, Pyrosequencing, and Melting Curve Analysis for the Detection of KRAS Mutations. J. Mol. Diagn..

[85] Mu W., Lu H.-M., Chen J., Li S., Elliott A.M. (2016). Sanger Confirmation Is Required to Achieve Optimal Sensitivity and Specificity in Next-Generation Sequencing Panel Testing. J. Mol. Diagn..

[86] Chen F., Dong M., Ge M., Zhu L., Ren L., Liu G., Mu R. (2013). The History and Advances of Reversible Terminators Used in New Generations of Sequencing Technology. Genom. Proteom. Bioinform..

[87] Raghavendra P., Pullaiah T., Raghavendra P., Pullaiah T. (2018). Chapter 7—Pathogen Identification Using Novel Sequencing Methods. Advances in Cell and Molecular Diagnostics.

[88] Clark M.J., Chen R., Lam H.Y.K., Karczewski K.J., Chen R., Euskirchen G., Butte A.J., Snyder M. (2011). Performance Comparison of Exome DNA Sequencing Technologies. Nat. Biotechnol..

[89] Arslan S., Garcia F.J., Guo M., Kellinger M.W., Kruglyak S., LeVieux J.A., Mah A.H., Wang H., Zhao J., Zhou C. (2024). Sequencing by Avidity Enables High Accuracy with Low Reagent Consumption. Nat. Biotechnol..

[90] de Ronne M., Boyle B., Torkamaneh D. (2025). AVITI as an Alternative to Illumina for Low-Cost Genome-Wide Genotyping. Genome.

[91] Lim M.H., Kim J., Tu Z.J., Cheng Y.-W. (2025). Comparison of Sequencing-by-Synthesis and Avidity Base Chemistry Next-Generation Sequencing Platforms in Identifying Somatic Variants of Hematological Malignancies. J. Appl. Lab. Med..

[92] Liu S., Obert C., Yu Y.-P., Zhao J., Ren B.-G., Liu J.-J., Wiseman K., Krajacich B.J., Wang W., Metcalfe K. (2024). Utility Analyses of AVITI Sequencing Chemistry. BMC Genom..

[93] Olsen T.R., Talla P., Sagatelian R.K., Furnari J., Bruce J.N., Canoll P., Zha S., Sims P.A. (2025). Scalable Co-Sequencing of RNA and DNA from Individual Nuclei. Nat. Methods.

[94] Galindo-González L., Pinzón-Latorre D., Bergen E.A., Jensen D.C., Deyholos M.K. (2015). Ion Torrent Sequencing as a Tool for Mutation Discovery in the Flax (*Linum usitatissimum* L.) Genome. Plant Methods.

[95] Shin S., Kim Y., Oh S.C., Yu N., Lee S.-T., Choi J.R., Lee K.-A. (2017). Validation and Optimization of the Ion Torrent S5 XL Sequencer and Oncomine Workflow for BRCA1 and BRCA2 Genetic Testing. Oncotarget.

[96] Feng Z., Zhang B., Ding W., Liu X., Yang D.-L., Wei P., Cao F., Zhu S., Zhang F., Mao Y. (2013). Efficient Genome Editing in Plants Using a CRISPR/Cas System. Cell Res..

[97] Trudsø L.C., Andersen J.D., Jacobsen S.B., Christiansen S.L., Congost-Teixidor C., Kampmann M.-L., Morling N. (2020). A Comparative Study of Single Nucleotide Variant Detection Performance Using Three Massively Parallel Sequencing Methods. PLoS ONE.

[98] Broothaerts W., Cordeiro F., Corbisier P., Robouch P., Emons H. (2020). Log transformation of proficiency testing data on the content of genetically modified organisms in food and feed samples: Is it justified?. Anal. Bioanal. Chem..

[99] Bassani N., Niedzwiecki A., Collotta A., Maretti M., Mazzara M., Kreysa J. (2013). Comparative Testing Report on the Detection and Quantification of GM Events in Compound Feedstuff—Comparative Testing Round: ILC-EURL-GMFF-CT-02/12.

[100] Broothaerts W., Beaz H.M.R., Buttinger G., Corbisier P., Cubría R.M., Dehouck P., Emteborg H., Maretti M., Robouch P. (2022). Determination of GM Soybean MON89788 in Meat Paté (T1) and GM Maize T25 in Maize Flour (T2).

[101] Duan Y., Pi Y., Li C., Jiang K. (2021). An Optimized Procedure for Detection of Genetically Modified DNA in Refined Vegetable Oils. Food Sci. Biotechnol..

[102] Lee S.-I., Kim N.-S. (2014). Transposable Elements and Genome Size Variations in Plants. Genom. Inf..

[103] Heinz S., Neusius D., Eckermann K.N., Pietsch K., Guertler P. (2025). Development and In-House Validation of Two Real-Time PCR Methods for the Detection of Genome-Editing Events in Soybean FAD2 Gene Variants. J. Consum. Prot. Food Saf..

[104] Wang M., Liu X., Yang J., Wang Z., Wang H., Wang X. (2022). CRISPR/Cas12a-Based Biosensing Platform for the on-Site Detection of Single-Base Mutants in Gene-Edited Rice. Front. Plant Sci..

[105] Ren Q., Sretenovic S., Liu S., Tang X., Huang L., He Y., Liu L., Guo Y., Zhong Z., Liu G. (2021). PAM-Less Plant Genome Editing Using a CRISPR–SpRY Toolbox. Nat. Plants.

[106] Walton R.T., Christie K.A., Whittaker M.N., Kleinstiver B.P. (2020). Unconstrained Genome Targeting with Near-PAMless Engineered CRISPR-Cas9 Variants. Science.

[107] Su Z., Wang X., Chen X., Ding L., Zeng X., Xu J., Peng C. (2024). Novel CRISPR/SpRY System for Rapid Detection of CRISPR/Cas-Mediated Gene Editing in Rice. Anal. Chim. Acta.

[108] Chhalliyil P., Ilves H., Kazakov S.A., Howard S.J., Johnston B.H., Fagan J. (2020). A Real-Time Quantitative PCR Method Specific for Detection and Quantification of the First Commercialized Genome-Edited Plant. Foods.

[109] Weidner C., Edelmann S., Moor D., Lieske K., Savini C., Jacchia S., Sacco M.G., Mazzara M., Lämke J., Eckermann K.N. (2022). Assessment of the Real-Time PCR Method Claiming to Be Specific for Detection and Quantification of the First Commercialised Genome-Edited Plant. Food Anal. Methods.

[110] Fraiture M.-A., D’aes J., Gobbo A., Delvoye M., Meunier A.-C., Frouin J., Guiderdoni E., Deforce D., De Vogelaere C., De Keersmaecker S.C.J. (2025). Genetic Fingerprints Derived from Genome Database Mining Allow Accurate Identification of Genome-Edited Rice in the Food Chain via Targeted High-Throughput Sequencing. Food Res. Int..

[111] Pallarz S., Fiedler S., Wahler D., Lämke J., Grohmann L. (2023). Reproducibility of Next-Generation-Sequencing-Based Analysis of a CRISPR/Cas9 Genome Edited Oil Seed Rape. Food Chem. Mol. Sci..

[112] Broll H., Bendiek J., Braeuning A., Eckermann K.N., Gebhardt A., Grohmann L., Keiss N., Lämke J., Mankertz J., Schenkel W. (2025). Current Status and Trends in the Analysis of GMO and New Genomic Techniques. J. Consum. Prot. Food Saf..

[113] Fraiture M.-A., D’aes J., Guiderdoni E., Meunier A.-C., Delcourt T., Hoffman S., Vandermassen E., De Keersmaecker S.C.J., Vanneste K., Roosens N.H.C. (2023). Targeted High-Throughput Sequencing Enables the Detection of Single Nucleotide Variations in CRISPR/Cas9 Gene-Edited Organisms. Foods.

[114] Burns M., Nixon G. (2023). Literature Review on Analytical Methods for the Detection of Precision Bred Products. FSA.

